# C-Shaped Canal in Second Molar of Mandible among Cases of Cone Beam Computed Tomography in Tertiary Care Centres: A Descriptive Cross-sectional Study

**DOI:** 10.31729/jnma.6722

**Published:** 2021-07-31

**Authors:** Neera Joshi, Suraj Shrestha, Sunanda Sundas, Kranti Prajapati, Sharada Devi Wagle, Archana Gharti

**Affiliations:** 1 Department of Conservative Dentistry and Endodontics, People's Dentai College and Hospital, Kathmandu, Nepal; 2 Department of Paediatric and Preventive Dentistry, People's Dental College and Hospital, Kathmandu, Nepal

**Keywords:** *cone beam computed tomography*, *C-shaped canal*, *mandibular second molar*, *root canal*

## Abstract

**Introduction:**

C-shaped canal configuration is mostly found in the mandibular second molar. The morphological characteristic of a C-shaped canal is the presence of a fin or web connecting the individual canal, making it difficult for cleaning, shaping, and obturation. The objective of this study was to find out the prevalence of C-shaped canal in mandibular second molar among cases of Cone Beam Computed Tomography in tertiary care centres.

**Methods:**

The descriptive cross-sectional study was conducted in the department of conservative dentistry and endodontics of tertiary care centres from 20th June 2020 to 20th December 2020 after receiving ethical approval from the Nepal Health Research Council on 19 June 2020. Cone beam computed tomography images of 199 mandibular second molars with completely formed roots were used. Teeth with orthodontic braces, root resorption, root canal filling, and post were excluded from the study. The research was conducted taking a tooth as a unit. Convenience sampling was done. Statistical analysis was done by using Statistical Package for Social Sciences version 16. Point estimate at 95% Confidence Interval was calculated along with frequency and proportion for binary data.

**Results:**

The prevalence of C-shaped canal according to this study is 25 (12.6%) (7.99-17.21 at 95% Confidence Interval).

**Conclusions:**

The findings of the study conclude that C-shaped configuration is quite frequent in mandibular second molar among cases of Cone Beam Computed Tomography. A careful preoperative radiographic evaluation may be helpful for diagnosing C-shaped configuration prior to root canal treatment.

## INTRODUCTION

C-shaped canal configuration is commonly detected in mandibular molar and is named so because of its 'C' shaped cross-sectional appearance.^1^ Predicting and negotiating C-shaped canal is challenging therefore treatment protocol must be modified.^2^ There is higher prevalence of this configuration in Asian population as compared to other populations. Hence it is essential to know the difference between ethnic groups to facilitate effective treatment plan.^3^

Various methods have been used to determine root canal morphology including tooth sectioning, dye infiltration, staining, clearing method, radiography, computed tomography (CT), micro-CT, cone beam computed tomography (CBCT).^4^ CBCT aids in accurate identification and analysis of such canal's configuration.^5^

Moreover, there has not been any study done regarding determination of C-shaped canal using CBCT in our country.

The objective of this study is to find out the prevalence of C-shaped canal in mandibular second molar among cases of Cone Beam Computed Tomography in tertiary care centres.

## METHODS

A descriptive cross-sectional study was conducted in the department of conservative dentistry and endodontics, Peoples Dental College and Hospital, Sorakhutte, Dental Imaging Centre, Uttardokha and Himal Dental Hospital, Dhumbarahi over a period of seven months from 20 June 2020 to 20 December 2020 after receiving ethical clearance from Nepal Health Research Council on 19 June 2020 (Reference number 2716).

Patients between the ages of 20 to 70 years requiring CBCT were taken. The CBCT images were taken as part of the routine examination, diagnosis and treatment planning. The CBCT image including at least one permanent mandibular second molar with completely formed roots in scan was selected. Teeth with orthodontic braces, root resorption, root canal filling and post were excluded from the study. Convenience sampling technique was used to collect the sample. Informed written consent was obtained from all the patients whose CBCT images were taken in this study. Research was conducted taking a tooth as a unit. The Sample size was calculated using the formula,

n = Z^2^ × p × (1 - p) / e^2^

  = (1.96)^2^ × 0.39 × 0.61 / (0.07)^2^

  = 187

Where,

n = required sample size
Z = 1.96 at 95% Confidence Interval
p = prevalence of c-shaped canal from previous study, 39%^5^
q = 1-p
e = margin of error, 7%

However, CBCT images of 199 permanent mandibular second molars were taken into the study.

The images were viewed in a dark setting, free of outside distractions, using laptop. The CBCT images were adjusted and analysed using the Galileos Viewer software (Dentsply Sirona, Charlotte) for images of Himal Dental Hospital and Peoples Dental College; and Planmeca Romexis software (Planmeca, Finland) for images of Dental Imaging Centre. The images were analysed in axial, sagittal and coronal view concurrently by two endodontists. Both the observers examined the CBCT images of mandibular second molar for the presence of C-shaped canal configuration. The images with C-shaped configuration were analysed in axial view at the level of the canal orifice, coronal third portion (from the orifice to the coronal 1/3 point of canal length), middle third portion (from the coronal 1/3 point of canal length to the apical 1/3 point of canal length), and apical third portion (from the apical 1/3 point of canal length to the apical foramen) and classified according to Fan, et al.^5^ The images were analysed by two Endodontists, and if there were disagreement then third Endodontist assisted in making the decision.

**Figure 1:**
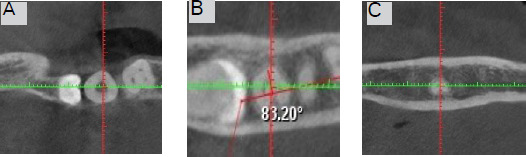
C-shaped configuration in different level of root canal: A) Coronal third, B) Middle third, C) Apical third.

The C-shaped canal configuration was classified at each level of the root according to Fan, et al.^5^ (Figure 1).

C1: continuous C-shaped canal
C2: semi-colon shaped because of discontinuation of the 'C' outline, but either angle a or b should be no less than 60°
C3: two (C3a) or three (C3b) separate canals and both angles a and b less than 60°
C4: single round or oval canal and
C5: no canal lumen

Statistical analysis was done by using Statistical Package for Social Sciences version 16. Point estimate at 95% Confidence Interval was calculated along with frequency and proportion for binary data.

## RESULTS

This study was conducted through imaging of a total of 199 mandibular second molars. Out of total 199 second molars, C-shaped canals were found in 25 (12.6%) (7.99-17.21 at 95% Confidence Interval) molars (Table 1). Among these, 108 (54.27%) teeth images were of male and 91 (45.72%) were of female.

**Table 1:** Frequency distribution of C-shaped canals in mandibular second molar (n=199).

C-shaped canals	Present	Absent
	n (%)	n (%)
	25 (12.6)	174 (87.4)

Among the total males, 11 (10.2%) of them had C-shaped canals while among the total females, 14 (15.4%) of them had this configuration. Also, among the total left and right mandibular molars, 14 (13.9%) and 11 (12.2%) of teeth had C-shaped canals respectively (Table 2).

**Table 2:** Frequency of C-shaped canals in mandibular second molars by sex and tooth position.

Sex	
Male	Female
**(n = 108)**	**(n = 91)**
11 (10.2)	14 (15.4)
**Tooth position**	
**Left**	**Right**
**(n = 101)**	**(n = 98)**
14 (13.9)	11 (12.22)

The cross-sectional shape of C-shaped canal at the orifice, coronal and middle level of the root, were mostly an uninterrupted C-shape (C1) while at the apical level the C2 and C4 shapes were most common. The C3a shape was not seen at orifice level and was least 1 (4%) common at coronal and middle level while C1 shape was least 2 (8%) common at the apical root level (Table 3).

**Table 3:** Cross-sectional canal shape of C-shaped canal at different levels (n=25).

Root level	C1 n (%)	C2 n (%)	C3a n (%)	C3b n (%)	C4 n (%)
Orifice	14 (56)	3 (12)	0	2 (8)	6 (24)
Coronal	10 (40)	8 (32)	1 (4)	3 (12)	3 (12)
Middle	9 (36)	8 (32)	1 (4)	4 (16)	3 (12)
Apical	2 (8)	8 (32)	4 (16)	3 (12)	8 (32)

## DISCUSSION

C-shaped canal anatomy was first reported by Cooke and Cox in 1979. The most widely accepted theory for the formation of this unusual ribbon shaped canal is the failure of Hertwig's epithelial root sheath to fuse either at the buccal or at the lingual root surface.^1^ Teeth with C-shaped canals possess several interconnections and ramifications that act as bacterial niches which are difficult to debride and disinfect.^6^

The mandibular second molar has high frequency for C-shaped configuration. Moreover, the prevalence in Asian population ranges from 2.7-45.5% in mandibular second molars.^4^ In the literature, the C-shaped canal was most common in a Korean subpopulation, with a 31-45% prevalence.^7,8^ The Chinese population also presented high prevalence of C-shaped canals, as reported by Yang, et al., Zheng Q, et al. and Zhang, et al. which is 32%, 39%, and 29% respectively. However, in Caucasian populations C-shaped configuration was not common as in Asian population. Cooke and Cox, Weine and Cimilli, et al. reported a prevalence of 2.7%, 7.6% and 8.1% of C-shaped canals, respectively in Caucasian populations.^9,10^

The CBCT is a non-invasive, non-destructive technique that provides high resolution image with low dose of radiation.^11^ It is highly accurate in identifying root canal systems as the modified canal staining and tooth clearing technique.^12^ In this study, two endodontists evaluated the root canal morphology of the mandibular second molar. This increased the reliability of the study. Similar method for image evaluation was used in a study done by Tian, et al.^13^ In this study Fan's classification (modified Melton's method) was used to classify C-shaped configuration. This classification is anatomic and is represented in axial cross section of tooth which is easy to observe in CBCT axial view. Same classification is used in similar study done by Kim, et al.^2^ The C-shaped canal configuration can vary along the root depth. Therefore, in this study, the cross-sectional canal shape was analyzed at four root level i.e., canal orifice, coronal, middle and apical third of the root.

The prevalence of C-shaped canals in this study is 12.6% which is in accordance with the study done by Vikram, et al.^14^ In the present study the prevalence of C-shaped canal among males is 11 (10.2%) while among females is 14 (15.4%). It has been concluded in previous studies that gender does not have a significant influence on the prevalence of C-shaped morphology.^5^ However, the result of the present study is in accordance with Martins, et al. who reported that women presented a higher prevalence.^15^ In this study, majority of the canals cross section demonstrated an uninterrupted C-shape (C1 category) at the orifice, coronal and middle level of the root, while C3 and C4 types increased at the apical region. This indicated that the continuous C1 category has a high possibility of dividing into two or three canals in the middle and apical regions. This result was not in accordance with Vikram, et al. who reported that at the apical level the C2 and C4 types were most common.^14^ The C3a shape was not seen at orifice level and was least 1 (4%) common at coronal and middle level while C1 shape was least 2 (8%) common at the apical root level.

## CONCLUSIONS

The findings of the study conclude that C-shaped configuration is quite frequent in mandibular second molar among cases of Cone Beam Computed Tomography in comparison to the study done in similar settings. Since C-shaped canal configuration is challenging, the clinician must be aware of diagnosing this configuration prior to initiating the treatment in order to increase the success rate of the treatment. The CBCT analysis is clinically effective method for determining root and canal morphology when conventional intraoral radiograph views produce limited information.
